# Oral Administration of Probiotic Bacteria Alleviates Tau Phosphorylation, Aβ Accumulation, Microglia Activation, and Memory Loss in 5xFAD Mice

**DOI:** 10.3390/brainsci14030208

**Published:** 2024-02-23

**Authors:** Yeong Jin Kim, Bo-Ram Mun, Kyu Yeong Choi, Won-Seok Choi

**Affiliations:** 1 School of Biological Sciences and Technology, College of Natural Sciences, College of Medicine, Chonnam National University, Gwangju 61186, Republic of Korea; vitalogy1032@gmail.com (Y.J.K.); boram5490@gmail.com (B.-R.M.); 2 Gwangju Alzheimer’s and Related Dementia Cohort Research Center, Chosun University, Gwangju 61452, Republic of Korea; khaser@gmail.com; 3 Kolab, Inc., Gwangju 61436, Republic of Korea

**Keywords:** Alzheimer’s disease, tau, amyloid beta, microglia, probiotics

## Abstract

The gut–brain axis (GBA) plays a significant role in various neurodegenerative disorders, such as Alzheimer’s disease (AD), and the gut microbiome (GM) can bidirectionally communicate with the brain through the GBA. Thus, recent evidence indicates that the GM may affect the pathological features and the progression of AD in humans. The aim of our study was to elucidate the impact of probiotics on the pathological features of AD in a 5xFAD model. Probiotics (*Bifidobacterium lactis*, *Levilactobacillus brevis*, and *Limosilactobacillus fermentum*) were orally administered in 5xFAD mice to modify the GM composition. Additionally, freeze-dried food containing phosphatidylserine was used as the positive control. Behavioral pathogenesis was assessed through the cross maze and Morris water maze tests. Our findings revealed that probiotic administration resulted in significant improvements in spatial and recognition memories. Furthermore, the neuroprotective effects of probiotics were substantiated by a reduction in amyloid-β accumulation in critical brain regions. Microglial activation in 5xFAD mice was also attenuated by probiotics in the hippocampus and cerebral cortex. Moreover, elevated tau phosphorylation in 5xFAD mice was ameliorated in the probiotics-treated group. The results highlight the potential use of probiotics as a neuroprotective intervention in AD.

## 1. Introduction

Alzheimer’s disease (AD) is a chronic, progressive neurodegenerative disease that affects brain functions, including memory and cognition. The core pathophysiology of AD has been postulated as the intraneuronal aggregation of hyperphosphorylated tau, a microtubule-associated protein, which leads to the formation of neurofibrillary tangles, and the interstitial aggregation of insoluble forms of the amyloid-β (Aβ) peptide, which results in neuritic plaques [1]. Less than 1% of cases are characterized by the familial type of AD, which is associated with autosomal dominant inheritance, Aβ overexpression, and onset often before the age of 65 years [2]. In the sporadic form of AD, both genetic and environmental risk factors play a role. Plaque formation in the brain has toxic effects on neurons and occurs due to the overproduction or decreased clearance of Aβ [3]. Additionally, substantial evidence suggests that neuroinflammation plays a key role in the pathophysiology of AD. Specifically, the accumulation of activated microglia around damaged areas is a hallmark of neuroinflammation in AD. Moreover, microglia can be activated by pathogens and improperly deposited proteins such as Aβ in AD [4].

The brain interacts with the gut either through the nervous system or through chemical substances that cross the blood–brain barrier (BBB). The gut microbiota (GM) produces amino acids and monoamines that, through the lymphatic and vascular systems, reach the central nervous system (CNS) where they can affect its activity [5]. Additionally, aging causes substantial changes in the content and function of the gut–brain axis (GBA), which can have an impact on health and age-related disorders [6]. Emerging evidence suggests the GBA’s role in neurodegenerative disease progression through diverse pathways [7]. Bacterial metabolites contribute to immune and metabolic changes, potentially increasing intestinal and blood–brain barrier permeability, modulating inflammatory responses, and influencing microglial maturation, which is crucial for CNS homeostasis and regulating inflammation. Notably, research has identified a correlation between gut dysbiosis and the aggregation of Aβ, the formation of tau proteins, and the onset of neuroinflammation and oxidative stress, all of which are implicated in AD [8].

Recent studies indicate that the consumption of specific probiotics can ameliorate a range of diseases, including sepsis, cancer, and neurodegeneration, via antioxidant and anti-inflammatory pathways [9]. Clinical studies have provided strong evidence supporting potential interventions of probiotics for neurodegenerative diseases. Of these strains, lactic acid bacteria (LAB), such as *Lactobacillus* and *Bifidobacterium*, are the most widely utilized probiotics [10]. According to a preclinical study, probiotics may improve cognitive function in animal models of cognitive impairment [11]. It has been demonstrated that mixed cultures of LAB and Bifidobacterium strains exert a synergistic effect on host health, leading to the enhanced production of short-chain fatty acids and modulation of the immune response [12]. Thus, it is important to investigate the possible therapeutic advantages of probiotics on brain health in degenerative diseases like AD [13].

In this study, we investigated the effect of probiotics on amyloid-induced pathology in an AD model. The 5xFAD mice, containing APP and presenilin mutants, are commonly used as an AD model, constituting approximately 10% of all AD studies that employ an animal model [14]. These mice develop amyloid pathology, with plaques appearing in the brain between 2 to 4 months of age [15]. In contrast, the 3xTG mouse model, which contains APP, presenilin, and tau mutants, exhibits a much slower disease progression and amyloid deposition starting around 6 months. For our study, we used 4-month-old 5xFAD mice and investigated the effect on tau, Aβ, and microglial activity.

## 2. Materials and Methods

### 2.1. Animals

Four-month-old 5XFAD (APP K670N/M671L [Swedish], APP I716V [Florida], APP V717I [London], PSEN1 M146L, and PSEN1 L286V; Jackson Laboratory, Bar Harbor, ME, USA) mice and wild-type (WT) littermates were housed under a regular 12 h light/12 h dark cycle at room temperature (20–25 °C) and supplied with food and water [16]. For the experiment, each mouse (WT: male *n* = 7, female n = 8; 5xFAD: male n = 16, female n = 16) was placed into an individual cage. All protocols for the animal experiments were approved by the Institutional Animal Care and Use Committee of Chonnam National University (CNU IACUC-YB-2022-154; CNU IACUC-YB-2023-156) [17].

### 2.2. Probiotics and Phosphatidylserine Supplementation

The mice were divided into four groups: 5xFAD, 5xFAD/Probiotics, 5xFAD/Phosphatidylserine (PS), and WT. Starting from 4 months of age, the probiotics group was supplemented daily with 8 × 10^7^ CFU of probiotics (*Bifidobacterium lactis* KL101, *Limosilactobacillus fermentum* KL271, and *Levilactobacillus brevis* KL251; Kolab, Inc., Gwangju, Republic of Korea) in 8 mL sterilized water for 3 months. The probiotics were prepared as described with minor modifications [9]. The bacterial cultures were stored at 4 °C and used in their live state. PS significantly improved cognitive function when administered to AD patients [18]. Several studies confirmed the efficacy of PS in AD patients and an animal model [19,20,21]. In the rodent model, the effective dose was 15–30 mg/kg and we used 21mg/kg daily in this study. The PS group, as a positive control, was fed freeze-dried food containing PS for 3 months.

### 2.3. Open Field Test

After 2 months of probiotics supplementation, the mice were subjected to behavior tests. Locomotor activity was measured in an open-field test as described [22]. Each mouse was placed in the center of the arena (40 × 40 × 40 cm) and allowed to freely explore for 5 min for prehabituation. In the main test, the movement of each mouse was recorded for 20 min and analyzed using ANY-maze software ver. 6.32 (Stoelting, Wood Dale, IL, USA).

### 2.4. Cross Maze

Each mouse was placed in a cross-shaped maze with four arms and monitored. The duration of the examination period was 10 min. The rate of spontaneous alternation was determined using the following formula: actual alternation/(possible alternation [total number of arm entries] − 3) × 100 (%) [17].

### 2.5. Morris Water Maze

The Morris water maze (MWM) test utilized a circular, open pool, which was filled with opaque water, with a diameter of 114 cm [23]. The water became opaque using non-toxic paint and the temperature was maintained at ~24 °C. A hidden platform (17 × 10.5 cm) was strategically positioned in the target quadrant below the water surface [24]. The mice were then subjected to the acquisition and probe phases. In the acquisition phase, each mouse underwent training, which involved four trials per day for 4 days [25]. During each trial, the mice were given a 60 s time limit to swim, and the trial concluded as soon as they reached the platform. Following the completion of each trial, the mice were allowed to remain on the platform for 20 s. After finishing the 4-day acquisition phase, a probe test was performed without the platform, lasting for 90 s. The movement of each mouse was then recorded, and time spent in the designated target quadrant and the latency to reach the platform were analyzed using ANY-maze software.

### 2.6. Immunohistochemistry

The animals were euthanized after a 3-month period of receiving treatments. Subsequently, their brains were extracted, fixed in a 4% paraformaldehyde solution, and then soaked in phosphate-buffered saline (PBS) with 30% sucrose overnight. The brain tissues were then embedded in optimum cutting temperature (OCT) freezing media, sliced (30 μm thickness), and mounted on glass slides. Brain sections were permeabilized using 0.15% Triton X-100 in PBS (PBST) for 30 min and then washed with PBS. The sections were then incubated with 3% bovine serum albumin (BSA) and 3% goat serum in PBS with 0.1% Triton X-100 for 30 min. The primary antibodies, mouse anti-Aβ (6E10, 1:3000; BioLegend, San Diego, CA, USA) and rabbit anti-IBA-1 (1:3000; Wako, Richmond, VA, USA), were applied to the samples for 3 days at 4 °C. The sections were then washed with PBS and incubated with the secondary antibodies, Alexa Fluor 568 anti-mouse IgG or Alexa Fluor 488 goat anti-rabbit IgG (1:2000; Invitrogen, Waltham, MA, USA), overnight at 4 °C.

### 2.7. Quantification and Image Analysis

Fluorescence images were captured utilizing the EVOS M7000 (Invitrogen, Waltham, MA, USA) microscope. Subsequent quantification and analyses were carried out utilizing ImageJ software ver. 1.53q developed by the National Institutes of Health (NIH, https://imagej.net/ij/). The immunoreactivity of Aβ was calculated as the percent area of immunoreactive cells per slide (%). Regarding the microglia, the relative immunoreactivity was determined based on the stained slides. Images were taken using a Leica Stellaris 5 (Leica, Deerfield, IL, USA) confocal microscope to analyze microglial branches z-stacked at 1.5 µm intervals and compressed to about 15 images.

### 2.8. Microglia Morphology Analysis

A ramified cell is one that exhibits a complex network of processes originating from the cell body. Changes in microglia ramification indicate a microglial response to altered physiological conditions. ImageJ software (NIH) was consistently employed to process all photomicrographs, converting them into binary and skeletonized images. To analyze the microglial branches, we measured processes from a total of 10–20 microglia for each location in one mouse and summarized the number of branches and the total process length using ImageJ software. In addition to generating skeletonized images, we manually counted the cell bodies in each photomicrograph [26].

### 2.9. Protein Preparation

The brain cortex was collected in radioimmunoprecipitation assay (RIPA) lysis buffer composed of 25 mM Tris-HCl (pH 7.6), 0.01% Nonidet P-40, 150 mM NaCl, 1% sodium deoxycholate, 0.1% sodium dodecyl sulfate (SDS), 100 mM phenylmethylsulfonyl fluoride (PMSF), and protease inhibitors. These lysates were mechanically homogenized using a 26-gauge needle with 10 repetitions. Subsequently, the samples were centrifuged at 16,100 cfg for 30 min at 4 °C. RIPA lysis buffer was utilized in a volume of 200 µL for small tissue samples and 500 µL for larger tissues, such as the cortex. The protein concentration in the lysates was determined using the bicinchoninic acid (BCA) assay.

### 2.10. Western Blot

Lysates containing 35 µg of protein were separated on a 10% acrylamide gel through SDS-polyacrylamide gel electrophoresis (PAGE). The separated proteins were subsequently transferred onto polyvinylidene difluoride (PVDF) or nitrocellulose membranes (Amersham, Little Chalfont, UK). To prevent non-specific binding, the membranes were blocked using a blocking buffer composed of 5% skim milk in Tris-buffered saline (TBS) containing 0.1% Tween 20 (TBST) buffer for 30 min at room temperature. Following the blocking step, the membrane was washed four times with TBST for 5 min each time. Afterward, it was incubated with the primary antibody, mouse anti-PHF-1 (1:3000), in TBST overnight at 4 °C. The next day, the membrane was exposed to the goat anti-mouse HRP-conjugated secondary antibody (ENZO, Farmingdale, NY, USA) in TBST at a dilution of 1:10,000 at room temperature for 2 h. Finally, the immunoreactive bands were selectively detected using the enhanced chemiluminescence (ECL) reaction.

### 2.11. Statistical Analysis

Behavioral data, images, and Western blot data underwent analysis using a two-way analysis of variance (ANOVA) and one-way ANOVA, followed by post hoc analysis utilizing Tukey’s post hoc test. All presented data are expressed as the mean ± standard error of the mean (SEM). Statistical significance was determined with a threshold of *p* < 0.05, where values below this threshold were considered statistically significant.

## 3. Results

### 3.1. Probiotics Improve Recognition, Memory, and Spatial Memory in 5xFAD Mice

**Figure 1:**
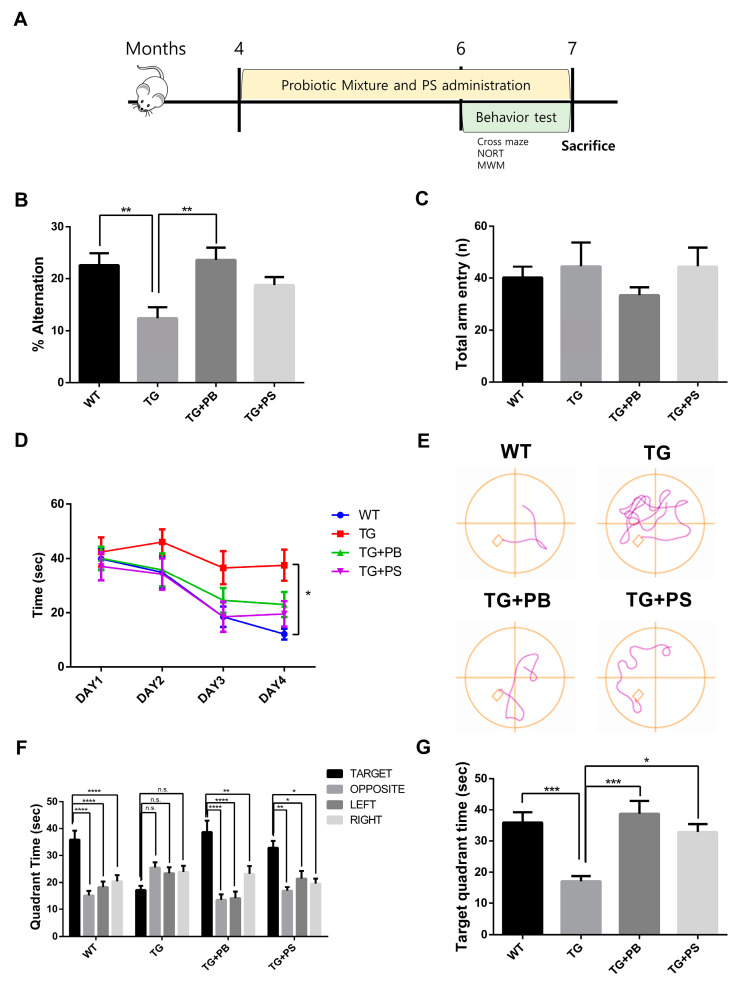
Probiotics alleviated the memory defect in 5xFAD mice. (**A**) The experimental procedure for the mice is displayed. (**B**,**C**) The cross maze and (**D**–**G**) Morris water maze were utilized to assess the learning and memory of mice. (**B**) In the cross maze test, the loss of the alternation rate was attenuated by probiotics, (**C**) but no changes in the number of total arm entries of the 5xFAD mice were seen. (**D**–**G**) Probiotics also diminished the defects in learning and memory in the Morris water maze test. (**D**) Escape latency during the learning phase. (**E**) Representative track plot of Day 4. (**F**) Time spent in each quadrant (seconds) during the probe phase. Treatment, F (3, 35) = 0.4372, *p* = 0.7278; Quadrant time, F (3, 105) = 16.58, *p* < 0.0001; Interaction Treatment × Quadrant time, F (9,105) = 5.365, *p* < 0.0001 was analyzed using two-way ANOVA, post hoc Tukey’s test. (**G**) Target quadrant time (seconds) in the probe phase. WT: Wild type, *n* = 13; TG: 5xFAD transgenic mice, *n* = 9; TG + PB: Probiotic-treated 5xFAD transgenic mice, *n* = 9; TG + PS: Phosphatidylserine-treated 5xFAD transgenic mice, *n* = 8. One-way analysis of variance (ANOVA) test; two-way ANOVA test; n.s, not significant; *, *p* < 0.05; **, *p* < 0.01; ***, *p* < 0.001; ****, *p* < 0.0001.

In this study, we investigated the hypothesis that probiotics enhance memory in animal models of AD, as suggested by a preclinical study [11,12]. To test this, we supplied a mixture of probiotic bacteria (*Bifidobacterium lactis*, *Levilactobacillus brevis*, and *Limosilactobacillus fermentum*) and phosphatidylserine (PS) as a positive control or the vehicle for 2 months before the behavior study (Figure 1A). PS improved the pathology and symptoms of AD by inhibiting neuroinflammation, increasing glucose metabolism in the brain, and normalizing N-Methyl-D-aspartate (NMDA) receptor activity [27]. Cognition and memory were evaluated in 5xFAD mice, an AD mouse model, using cross maze, novel object recognition, and MWM tests, while continuing to supply probiotics to the mice. In the cross maze test, the 5xFAD mice treated with probiotics or PS showed a significant improvement in their memory, represented by a higher alternation ratio, compared to that of the untreated 5xFAD mice. However, there was no change in the total arm entry (Figure 1B,C). Similarly, in the novel object recognition test, the 5xFAD mice treated with probiotics or PS exhibited enhanced memory, showing a higher preference index compared to that of the untreated 5xFAD mice, though the difference was not statistically significant (Supplemental Figure S1). Furthermore, in the MWM test to assess hippocampus-dependent spatial learning and memory, a notable increase in the memory (represented by decreased time spent in the target quadrant) was observed in the 5xFAD mice treated with probiotics or PS in the probe test as compared to that of the vehicle-treated 5xFAD mice (Figure 1D–G). These findings strongly suggest that probiotics can have a protective effect on the loss of spatial and recognition memories in the 5xFAD mouse model.

### 3.2. Probiotics Alleviated Aβ Accumulation in the Hippocampus and Cerebral Cortex of 5xFAD Mice

**Figure 2:**
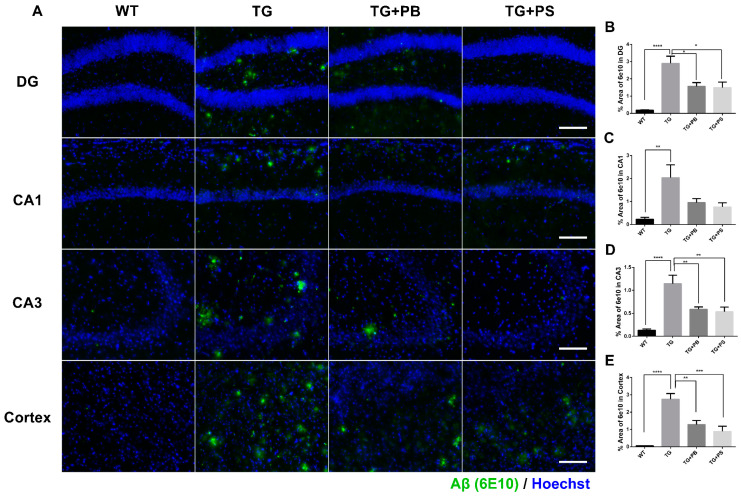
Probiotics decreased amyloid-β (Aβ) accumulation in the hippocampus and cerebral cortex in 5xFAD mice. (**A**) Representative immunohistochemistry images of the hippocampus and cerebral cortex. Scale bar = 100 μm. (**B**–**E**) Probiotics significantly decreased the Aβ level as quantified as the percent area of Aβ staining (6E10 positive) in the dentate gyrus (DG), Cornu Ammonis (CA) 1, CA3, and cortex in 5xFAD mice. Phosphatidylserine showed a similar protective effect. WT: Wild type, *n* = 6; TG: 5xFAD transgenic mouse, *n* = 6; TG + PB: Probiotics-treated 5xFAD transgenic mice, *n* = 6; TG + PS: Phosphatidylserine-treated 5xFAD transgenic mice, *n* = 5. One-way analysis of variance (ANOVA) test; *, *p* < 0.05; **, *p* < 0.01; ***, *p* < 0.001; ****, *p* < 0.0001.

A recent study found significant variations in the composition of the GM (which regulates the decline in cognitive function) and an increase in Aβ deposition in an AD model [28]. Therefore, we investigated the Aβ-positive area in the hippocampus and cerebral cortex to further evaluate the neuroprotective effects of probiotics. In the tissue staining, a significant decrease in the Aβ (6E10)-positive area was seen in 5xFAD mice that were treated with either probiotics or PS compared to that of the untreated 5xFAD mice (Figure 2). These findings provide compelling evidence of the neuroprotective effects of probiotics, especially in the hippocampus and cerebral cortex, thus highlighting their potential use as a treatment for AD.

### 3.3. Probiotics Reduced Microglial Activation in the Hippocampus and Cerebral Cortex in 5xFAD Mice

**Figure 3:**
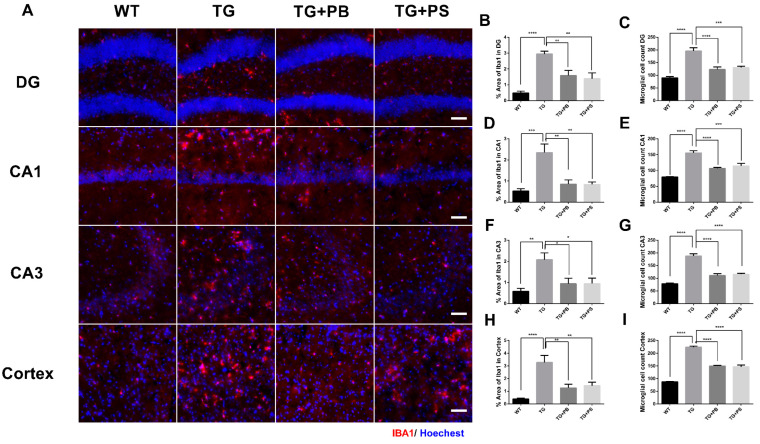
Activation of microglia in 5xFAD mice was reduced by probiotics. (**A**) Representative images of activated microglia (IBA-1) in the hippocampus and cerebral cortex. Scale bar = 100 μm. (**B**–**I**) Probiotics reduced the stained area of microglia and the number of microglia in the dentate gyrus (DG), Cornu Ammonis (CA) 1, CA3, and cortex in 5xFAD mice treated with either probiotics or phosphatidylserine, as compared to that of the vehicle-treated 5xFAD mice, and as quantified as the percent area of IBA-1 immunoreactivity. WT: Wild type, *n* = 6; TG: 5xFAD transgenic mouse, *n* = 6; TG + PB: Probiotics-treated 5xFAD transgenic mouse, *n* = 6; TG + PS: Phosphatidylserine-treated 5xFAD transgenic mouse, *n* = 5. One-way analysis of variance (ANOVA) test; *, *p* < 0.05; **, *p* < 0.01; ***, *p* < 0.001; ****, *p* < 0.0001.

Microglial activation is a prominent feature of AD [29] and the impact of the host GM on microglia homeostasis has been suggested [30]. We investigated the effects of probiotics on microglia by evaluating the intensity of the activated microglia in the hippocampus and cerebral cortex in 5xFAD mice. As a result, increased immunoreactivity and activated microglia were observed in the brains of 5xFAD mice (Figure 3). However, 5xFAD mice treated with either probiotics or PS showed a significant reduction in both the number and activity of microglia as compared to that of the vehicle-treated 5xFAD mice (Figure 3). These findings provide compelling evidence for the role probiotics play in alleviating microglial activation in the AD model.

### 3.4. Probiotics Suppressed Microglial Activation in 5xFAD Mice

**Figure 4:**
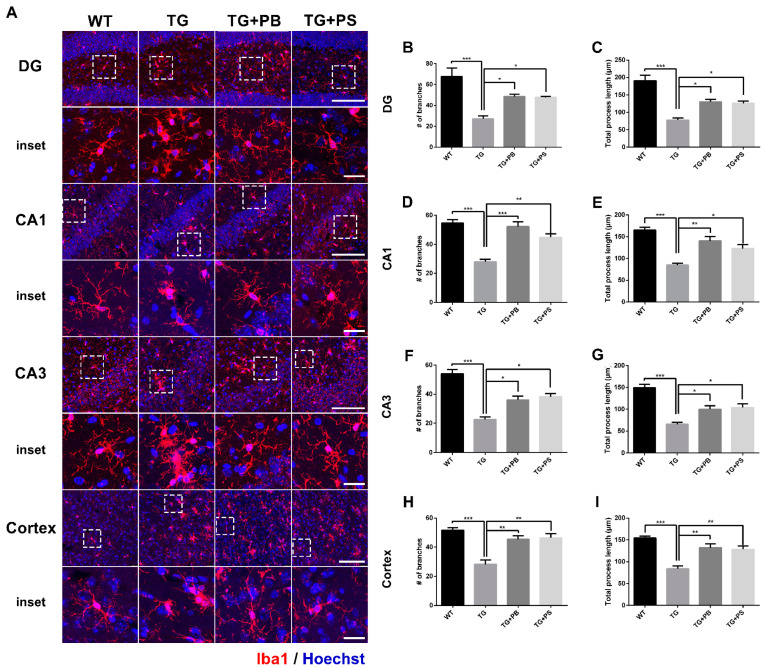
Probiotics suppressed microglial activation in the dentate gyrus (DG), Cornu Ammonis (CA) 1, and CA3 in the hippocampus and cerebral cortex of 5xFAD mice. (**A**) Representative images of microglial processes in the DG, CA1, and CA3 in the hippocampus and cerebral cortex. The inset indicates an enlarged area of the upper white dotted frame. Scale bar = 100 μm, inset scale bar = 20 μm. Our results demonstrate that the administration of probiotics and phosphatidylserine to 5xFAD mice resulted in increased numbers of branches and the total process length of microglia, as compared to that of the 5xFAD mice in the (**B**,**C**) DG, (**D**,**E**) CA1, and (**F**,**G**) CA3 in the (**H**,**I**) hippocampus and cerebral cortex. WT: Wild type, n = 6; TG: 5xFAD transgenic mouse, n = 6; TG + PB: Probiotics-treated 5xFAD transgenic mouse, n = 6; TG + PS: Phosphatidylserine-treated 5xFAD transgenic mouse, n = 5; One-way analysis of variance (ANOVA) test; *, *p* < 0.05; **, *p* < 0.01; ***, *p* < 0.001.

We further analyzed the detailed morphological modification of microglial activation using the microglial skeleton analysis method. Our results indicate that the number of branches and the total process length of the microglia were reduced in the hippocampus and cerebral cortex (Figure 4 and Figure S2) of 5xFAD mice. However, the administration of probiotics and PS attenuated these changes in 5xFAD mice (Figure 4). Taken together, these findings imply that the use of probiotics may efficiently suppress microglial activation in the AD mouse model.

### 3.5. Probiotics Reduce Phosphorylated Tau in the Cerebral Cortex in 5xFAD Mice

**Figure 5:**
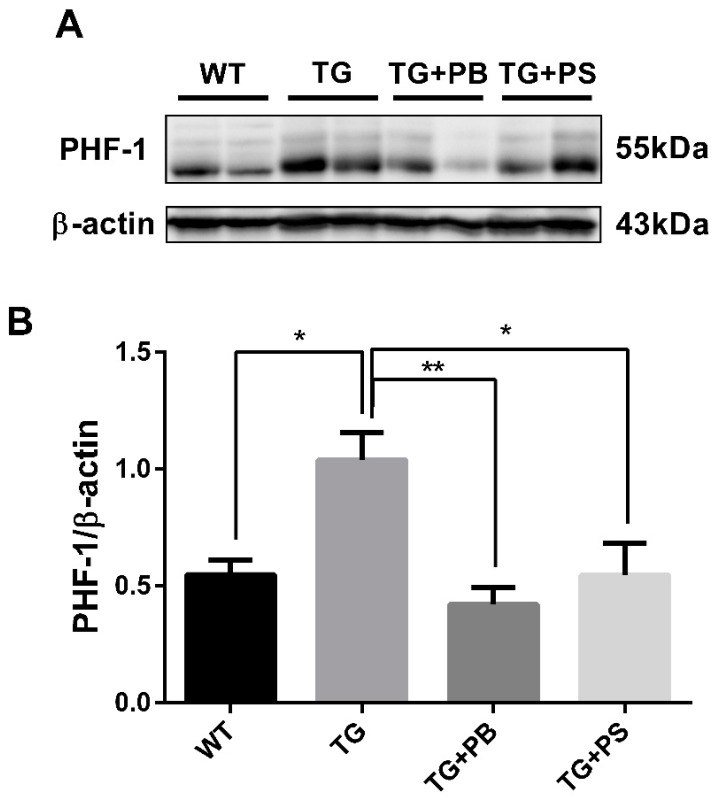
Probiotics reduce p-tau (s396, s404; PHF-1) in the cerebral cortex. (**A**) A representative Western blot of the cerebral cortex revealed that the level of phosphorylated tau (PHF-1) was significantly decreased in the 5xFAD group treated with probiotics when compared to that of the vehicle-treated 5xFAD group. A similar effect was observed in mice subjected to phosphatidylserine. (**B**) Immunoblot quantification of phosphorylated tau. WT: Wild type, n = 4; TG: 5xFAD transgenic mouse, n = 4; TG + PB: Probiotics-treated 5xFAD transgenic mouse, n = 4; TG + PS: Phosphatidylserine-treated 5xFAD transgenic mouse, n = 4. One-way analysis of variance (ANOVA) test; *, *p* < 0.05; **, *p* < 0.01.

Tau hyperphosphorylation is another distinct pathological feature in the brains of AD patients. Accumulating evidence suggests microglia play a role in the tau level and distribution in AD [31,32]. Recent studies have reported that microglial activation is an important factor that accelerates the aggregation and propagation of tau [32,33]. In this study, we observed the tau phosphorylation level using a phosphorylated tau-specific antibody. In the Western blotting assay, tau phosphorylation was elevated in 5xFAD mice, as compared to that of the WT mice (Figure 5 and Figure S3). However, it was significantly reduced in the 5xFAD group treated with probiotics, which was comparable with the effect of PS treatment (Figure 5). These findings strongly support the idea that probiotics have a beneficial effect in reducing tau phosphorylation in the brain of an AD mouse model.

Altogether, our data suggest that oral uptake of probiotics alleviated Aβ or tau pathology and memory loss in an AD model, which might be mediated by the attenuation of microglial activation.

## 4. Discussion

Currently, the existing treatments for AD are primarily focused on symptom management rather than halting or preventing the progression of the disorder. However, therapeutic approaches directly targeting Aβ or tau have had limited application up until now. Recently, significant research efforts have been focused on GM and GBA as alternative mechanisms to regulate AD. This approach showed a potential for the development of novel therapeutic interventions for AD [34]. Specifically, oral bacteriotherapy, which involves orally administering beneficial bacteria, is gaining recognition as a viable strategy for preventing and treating various disorders, including those related to the CNS [11,35,36], given that probiotics may enhance recognition and spatial memories in animal models of cognitive impairment. Therefore, we hypothesized that probiotics may have a therapeutic effect on 5xFAD mice. In our various behavioral tests, we found that probiotics improved recognition and spatial memories in 5xFAD mice.

In the previous report, oral supplementation with *Bifidobacterium breve* had a protective effect against memory impairment in an AD model [37]. The probiotics (*Bifidobacterium lactis*, *Levilactobacillus brevis*, and *Limosilactobacillus fermentum*) also improved LPS-induced cognitive impairment and memory loss in mice [9]. In this study, we used the same combination of live probiotic bacteria strains to investigate their therapeutic effects on AD. The probiotics improved the cognitive defects and memory loss and attenuated Aβ accumulation in 5xFAD mice. Additionally, the protective effect of the combined strains on Aβ and memory in this study was more pronounced than that of the single strain used previously. This suggests a beneficial effect of the combined use of probiotics. However, direct comparisons were not possible because the two studies used different AD models. Hence, further investigation comparing a single strain and multiple strains in a model would give direct evidence of the beneficial effect of combining multiple strains.

A recent study utilized WT and APP^swe/PS1ΔE9^ transgenic mice to investigate the role of dysbiosis in AD using several methods. The investigation results revealed that dysbiosis induced heightened Aβ accumulation, significantly altered GM composition, and caused a decline in cognitive function, suggesting the importance of the GM in preventing Aβ burden in an AD model [38]. Consequently, our findings show that probiotics notably reduced Aβ accumulation and prevented cognitive/memory dysfunction in the AD model. Although Aβ has long been believed as the main pathological cause of AD, it has been consistently demonstrated that the accumulation and deposition of amyloid-β (Aβ) plaques do not exhibit a direct correlation with neuronal loss or cognitive decline [39,40,41]. Moreover, many individuals exhibit a substantial burden of amyloid plaques, as assessed through positron emission tomography (PET) scans, yet remain asymptomatic in terms of memory impairment [42]. On the other hand, the mean prevalence of amyloid positivity was 88% among the patients diagnosed with AD, meaning that significant cases of AD are not Aβ-dependent [43]. These brought up questions about the role of Aβ in AD. Nevertheless, the observed efficacy of recently approved anti-Aβ therapies, such as Lecanemab and Donanemab, in mitigating cognitive decline provides further evidence supporting the pathological involvement of amyloid-β (Aβ) in neurodegenerative processes [44].

Furthermore, inflammation-mediated pathways may trigger neurodegenerative diseases via the GBA. Chronic low-grade inflammation observed in older individuals may influence neuroinflammation by affecting glial cells, which may result in cognitive impairment [45]. In AD, neuroinflammation is driven by microglia [46], which can be modified to several morphological types. The hyperramified microglia are one type of activated microglia, which have an enlarged soma and thickened processes. The hyperramified microglia have been described in post-mortem AD, aged, and stressed human brains [47,48], which is possibly involved in stress-related synaptic modifications. On the other hand, dystrophic microglia are described with beading and fragmentation of the branches of the microglia [49]. Dystrophic microglia, which are observed in close proximity to Aβ deposits in the brains of postmortem AD patients, are hypothesized to represent a senescent phenotype of microglia [49,50]. In this condition, microglia lose their capacity to respond to chronic inflammatory stimuli and to perform their neuroprotective functions [47]. In a previous study, the microglia of 5xFAD mice had shorter processes and fewer branches compared to the microglia of WT mice and we observed similar microglial modifications [51]. In our data, probiotics suppressed the modifications of microglia, which can promote toxic activity and suppress the beneficial effects of microglia, leading to neuroinflammation in 5xFAD mice.

One defining pathological characteristic of the brains of patients with AD is the hyperphosphorylation of tau. In AD, patients’ brains have been shown to exhibit excessive tau phosphorylation. This impedes the normal attachment of tau to microtubules, leading to AD pathology. Recently, it was proposed that probiotic intake may affect tau phosphorylation [52]. In our findings, probiotics inhibited tau hyperphosphorylation. Specifically, probiotics may suppress lipopolysaccharide (LPS)-generating bacteria and LPS-induced inflammation, which stimulates tau phosphorylation [53].

In summary, the current findings support the potential use of probiotics as an innovative therapeutic strategy for treating AD. We demonstrated the protective effect of probiotics in 5xFAD mice through several memory tests. In the cross maze, novel object recognition, and MWM tests, the probiotic treatment attenuated memory impairment in 5xFAD mice. Moreover, the neuroprotective effects of probiotics in AD were supported by a decreased accumulation of Aβ in the hippocampus and cerebral cortex. Microglial activation was also attenuated in these regions and the group that received probiotics showed a reduction in tau phosphorylation. These findings suggest a protective effect of probiotics to reduce Aβ burden, tau pathology, and microglia-mediated inflammation in the AD model. Future studies will comprehensively identify and elucidate the mechanisms of probiotics in the context of AD.

## Data Availability

The data are not not publicly available due to the confidentiality issues but are available from the corresponding author on reasonable request.

## Supplementary Materials

The following supporting information can be downloaded at: https://www.mdpi.com/article/10.3390/brainsci14030208/s1, Figure S1: Recognition memory defect was alleviated by probiotics in 5xFAD mice; Figure S2: Skeletonized images of microglia; Figure S3: Additional Western blot used for the quantification of phosphorylated tau (PHF-1) in Figure 5B.
