# Supplementary material for: Oral Administration of Probiotic Bacteria Alleviates Tau Phosphorylation, Aβ Accumulation, Microglia Activation, and Memory Loss in 5xFAD Mice
Source: Brain Sci. 2024 Feb 23;14(3):208. doi: 10.3390/brainsci14030208 (PMC10968487; doi:10.3390/brainsci14030208)
Supplement: Supplementary file 1 [file brainsci-14-00208-s001.zip › brainsci-2878468-supplementary.pdf]

Oral administration of probiotic bacteria alleviates tau phosphorylation, Aβ accumulation, microglia activation, and memory loss in 5xFAD mice

Yeong Jin Kim [1], Bo-Ram Mun [1], Kyu Yeong Choi [2,3], and Won-Seok Choi [1*]

Supplemental method

Novel object recognition test

A novel object recognition test (NORT) was performed using the open field arena. In the training phase, two identical objects were placed in two neighboring corners and each mouse was located and allowed to explore the arena for 8 min. In the testing phase, one item was replaced with a new one in the same arena, and the mouse explored for 8 min. The preference index (%) was calculated as follows: the time spent exploring the novel object (n) / the total time spent exploring both the novel and familiar objects (n + f) [1].

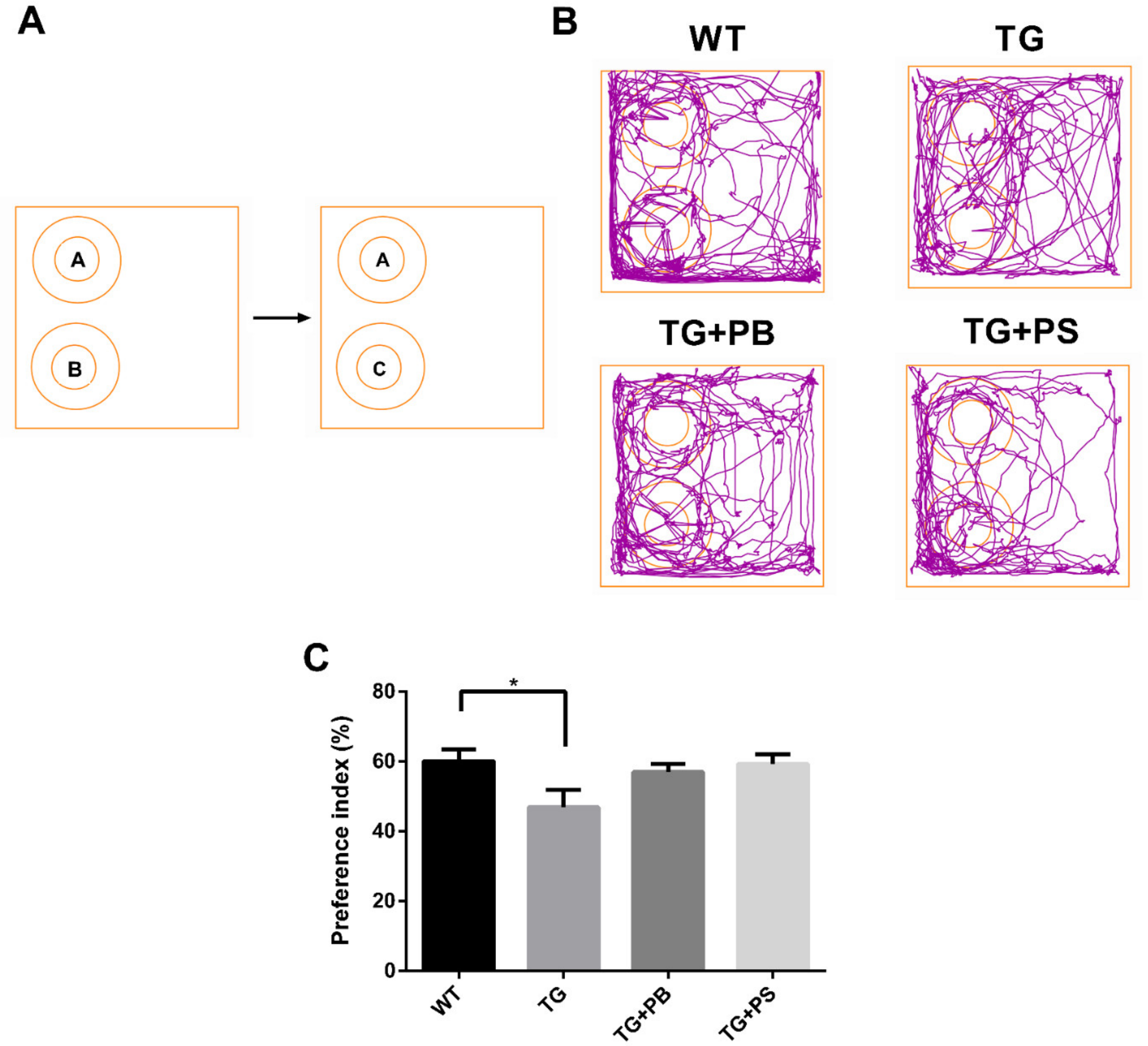

Supplemental Figure S1. Recognition memory defect was alleviated by probiotics in 5xFAD mice.

(A) The experimental scheme of Novel Object Recognition Test (NORT). (B) Representative track plots of NORT for old and new object at 24 hours after training. (C) Preference index (%) of NORT. WT: Wild type, n = 13; TG: 5xFAD transgenic mice, n = 9; TG+PB: Probiotic-treated 5xFAD transgenic mice, n = 9; TG+PS: Phosphatidylserine-treated 5xFAD transgenic mice, n = 8. One-way analysis of variance (ANOVA) test; Two-way ANOVA test; *, $p < 0.05$.

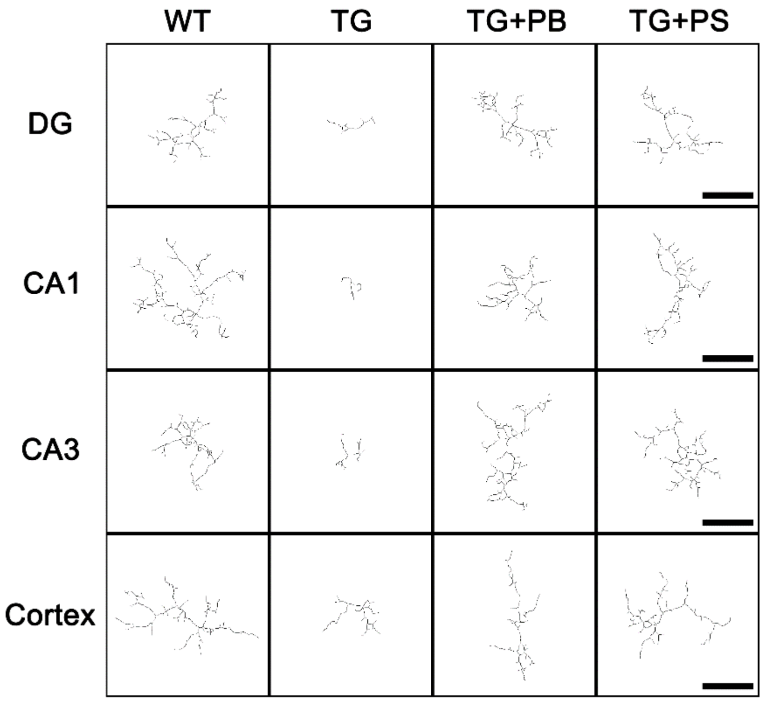

Supplemental Figure S2. Skeletonized images of microglia.

Representative skeletonized images of microglia (Figure 4) in hippocampus and cortex. Scale bar = 25um.

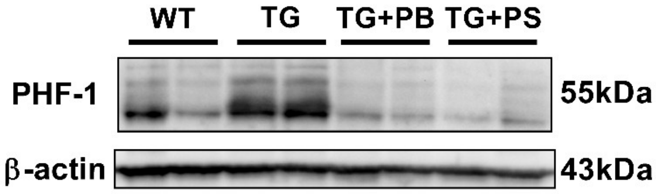

Supplemental Figure S3. Additional Western blot used for the quantification of phosphorylated tau (PHF-1) in Figure 5B.

Reference

1. Ramasamy, V. S.; Samidurai, M.; Park, H. J.; Wang, M.; Park, R. Y.; Yu, S. Y.; Kang, H. K.; Hong, S.; Choi, W. S.; Lee, Y. Y.; Kim, H. S.; Jo, J., Avenanthramide-C Restores Impaired Plasticity and Cognition in Alzheimer's Disease Model Mice. *Mol Neurobiol* **2020,** *57* (1), 315-330.
